# Comprehensive Pan-Cancer Analysis of TRPM8 in Tumor Metabolism and Immune Escape

**DOI:** 10.3389/fonc.2022.914060

**Published:** 2022-06-30

**Authors:** Wei Zhang, Xin-yu Qiao, Qian Li, Chun Cui, Chen-meng Qiao, Yan-qin Shen, Wei-jiang Zhao

**Affiliations:** ^1^ Cell Biology Department, Wuxi School of Medicine, Jiangnan University, Wuxi, China; ^2^ Department of Pathogen Biology, Guizhou Nursing Vocational College, Guiyang, China; ^3^ Department of Neurodegeneration and Neuroinjury, Wuxi School of Medicine, Jiangnan University, Wuxi, China

**Keywords:** TRPM8, pan-cancer, immune cell infiltration, tumor microenvironment, DNA repair

## Abstract

**Background:**

Transient receptor potential melastatin 8 (TRPM8) modulates tumor biology and sensitivity to treatment. The present study aimed to determine the part it plays in tumor immunity and physiology using pan-cancer analysis.

**Method:**

Data from the GTEx, CCLE, TISIDB, GSCA, cBioportal, and TCGA databases were collected using Estimate, Scanneo, and GSEA, and the associations between TRPM8 and prognosis, molecular subtypes, mutational burden, microsatellite instability, immune gene functions, and drug sensitivity were analyzed in 33 tumor types.

**Result:**

TRPM8 levels were found to be elevated in most tumors, particularly in solid tumors, with variations according to clinical stage. Mutation frequency was greatest in endometrial carcinoma. High levels of TRPM8 were linked to unfavorable prognosis, immune cell infiltration, and the tumor microenvironment, as well as correlating with abnormalities in the transcription levels of genes associated with immunity and DNA repair. TRPM8 was also linked to unfavorable patient outcomes and cancer-associated signaling.

**Conclusions:**

TRPM8 is strongly associated with tumor physiology and immunity. The Pan-Cancer analysis suggests the potential of TRPM8 as a treatment target or biomarker for determining the prognosis of a specific type of cancer.

## Introduction

The transient receptor potential melastatin 8 (TRPM8) protein is an ion channel mainly responsible for regulating Ca^2+^ permeabilization (1). When TRPM8 is activated, there is a Ca^2+^ influx into the cytoplasm, leading to the modulation of signaling pathways and ultimately of processes such as proliferation and migration (2, 3). Thus, the TRPM8 ion channel may regulate these processes in cancer cells by the enhancement of Ca^2+^-based intracellular signaling. TRPM8 is selectively expressed in organs and tissues, such as the prostate where it is found at relatively high levels. A certain amount of TRPM8 mRNA expression is also seen in the liver, root ganglia, and trigeminal ganglion cells (4). TRPM8 has also been found to be overexpressed in many tumors, including prostate, breast, colorectal, and pancreatic cancers, amongst others (5), suggesting that TRPM8 may have potential as a marker and drug target for cancer management.

Recent developments in high-throughput technologies and “omics” disciplines have opened new avenues in cancer research. The use of transcriptomics is well-known in cancer biology (6) and the recent application of deconvolution networks to transcriptomic data has allowed the extraction and analysis of immune gene expression patterns. In addition, the establishment of databases and the use of bioinformatics have enabled the assimilation of information on genes and their relations to cancer types (7). Pan-Cancer analysis allows the investigation of genetic connections across multiple tumor varieties, facilitating the analysis of mutations, expression, functions, and relations to patient prognosis (8).

There is a complex interplay between the tumor microenvironment (TME) and the immune system, with immune cells gaining access to and forming a significant part of the tumor surrounds (9). Although the TME is known to strongly affect cancer development, the dynamics of the process are poorly understood. Interactions between tumor and immune cells have been investigated for immunotherapeutic strategies such as immune checkpoint arrest, the harnessing of T cells, and vaccine development (10). Immunotherapy targeting immune and tumor cell interactions has proved efficacious (11). Several drugs that induce checkpoint arrest, targeting CTLA-4, PD-L1, and PD-1, have been developed (12, 13). It is possible that TRPM8 may also be an effective immunotherapeutic target; however, its role in cancer needs to be more precisely defined.

Considering the limited knowledge surrounding TRPM8 in cancer, we used multi-database Pan-Cancer analysis to investigate this question, in terms of the TME, genetic changes, prognosis, the tumor mutational burden (TMB), drug sensitivities, and microsatellite instability (MSI), especially in relation to immune genes, neoantigens, and checkpoints, in 33 cancer types. TRPM8 was found to modulate genes involved in DNA repair and methyltransferase activity, as well as regulate cancer-associated signaling pathways. The grade-dependent expression level of TRPM8 in several major tumor types was also evaluated using immunohistochemistry (IHC).

## Materials and Methods

### Tumor Samples

Information on genes and clinical characteristics for different tumors together with control tissues were acquired from the GTEx (https://gtexportal.org/) and TCGA (https://portal.gdc.cancer.gov/) databases. Information on cell lines was obtained from the CCLE database (https://portals.broadinstitute.org/). TIMER (https://cistrome.shinyapps.io/timer/) was used for information on immune infiltration scores. Data on the following tumors was acquired: ACC (Adrenocortical carcinoma); BLCA (Bladder Urothelial Carcinoma); BRCA (Breast invasive carcinoma); CESC (Cervical squamous cell carcinoma and endocervical adenocarcinoma); CHOL (Cholangiocarcinoma); COAD (Colon adenocarcinoma); COAD (Colon adenocarcinoma); READ (Rectum adenocarcinoma esophageal carcinoma); DLBC (Lymphoid Neoplasm Diffuse Large B-cell Lymphoma); ESCA (Esophageal carcinoma); GBM (Glioblastoma multiforme); HNSC (Head and Neck squamous cell carcinoma); KICH (Kidney Chromophobe); KIRC (Kidney renal clear cell carcinoma); KIRP (Kidney renal papillary cell carcinoma); LAML(Acute Myeloid Leukemia); LGG (Brain Lower grade Glioma); LIHC (Liver hepatocellular carcinoma); LUAD (Lung adenocarcinoma); LUSC (Lung squamous cell carcinoma); MESO (Mesothelioma); OV (Ovarian serous cystadenocarcinoma); PAAD (Pancreatic adenocarcinoma); PCPG (Pheochromocytoma and paraganglioma); PRAD (Prostate adenocarcinoma); READ (Rectum adenocarcinoma); SARC (Sarcoma); SKCM (Skin Cutaneous Melanoma); STAD (Stomach adenocarcinoma); STES (Stomach and Esophageal carcinoma); TGCT (Testicular germ cell tumors); THCA(Thyroid carcinoma); THYM (Thymoma); UCEC (Uterine Corpus Endometrial Carcinoma); UVM (Uveal Melanoma).

### Expression of TRPM8

TRPM8 levels in various tumors and controls were examined using “edgeR” in Bioconductor and the Kruskal Wallis test. The “ggplot” package in R was used to draw violin plots. For integrated analysis of TRPM8 differential expression in 33 cancers, the gene expression data of GTEx and TCGA were downloaded from the University of California Santa Cruz (UCSC) Xena database (https://xenabrowser.net/datapages/). Both profiles from raw RNA-Seq data were recomputed using the UCSC Xena project and converted to a format as log2(TPM+1).

### Genomic Alterations in TRPM8

Cancer genomic information was obtained from cBioPortal (http://www.cbioportal.org/) (14), including copy-number variations, mRNA levels, deep deletions, and missense mutations with unknown implications. UALCAN (http://ualcan.path.uab.edu) was utilized for examining DNA methylation.

### Prognostic Analysis of TRPM8

Univariate regression was performed to identify the relationships between TRPM8 level and patient outcomes and Kaplan-Meier curves were utilized to relate outcome to TRPM8 level. A bipartite technique was used to divide TRPM8 levels in tissue (tumor and control) into “high” and “low” groups for evaluation by univariate Cox regression. The “forestplot” package in R was used for visualization and PrognoScan (15) was used for meta-analysis of TRPM8 levels and the overall survival (OS) and disease-free survival (DFS) of patients.

### Subtype Analysis of TRPM8 in Pan-Cancer

The TISIDB database (http://cis.hku.hk/TISIDB/in-dex.php) (16) was used to investigate the link between TRPM8 and immune-associated genes and subtypes.

### Association of TRPM8 With the Immune Microenvironment

The presence and degree of immune infiltration, measured by the immune and stromal scores, are used to predict sentinel lymph node status and outcomes. The correlations between these scores and TRPM8 were determined, with significance levels of p < 0.05 and R > 0.20.

### Relationship Between TRPM8 and Immune-Related Neoantigens and Checkpoint Genes

Scanneo was used to detect neoantigens and the products of mutated genes. This predicts the binding affinities of proteins using short (8-11 residues) epitopes, with low scoring proteins classified as neoantigens, which are then ranked. The association between TRPM8 levels and neoantigen numbers per tumor was determined. TRPM8 levels were also associated with over 40 immune checkpoint-associated genes, with significance set at p < 0.05 and R > 0.20.

### Association of TRPM8 With TMB and MSI

The TMB is an indication of mutation numbers in a cancer cell. The MSI represents a predisposition to mutation caused by faulty DNA mismatch repair (MMR). Correlations between TRPM8 levels and TMB and MSI were assessed by Spearman’s rank correlation analysis.

### Association of TRPM8 With DNA MMR Genes and Methyltransferases

Impaired MMR leads to increased numbers of somatic mutations. TCGA TRPM8 levels were correlated with those of five MMR genes, namely, MLH1, MSH2, MSH6, PMS2, and EPCAM. DNA methylation modulates gene expression and is conducted by methyltransferases. TRPM8 levels were correlated with those of four methyltransferases with significance assessed as above. The R package “ggplot” was utilized to visualize the data.

### Protein-Protein Interaction (PPI) Network Analysis

PPI networks of TRPM8 and its interactors were created with the STRING (http://string.embl.de/) (17, 18) and GeneMANIA (http://www.genemania.org) (19) databases.

### Gene Set Enrichment Analysis

Gene set enrichment analysis (GSEA) was used to investigate TRPM8 in relation to “biological process”, “molecular function”, or “cellular component” (20) using the Kyoto Encyclopedia of Genes and Genomes (KEGG) and MsigDB (21) databases, using the Hallmark gene set. |NES| > 1, p < 0.05, and FDR < 0.25 were set as thresholds for GSEA.

### Drug Sensitivity Analysis

The influence of TRPM8 on drug susceptibility was assessed using the GSCA (Gene Set Cancer Analysis) (http://bioinfo.life.hust.edu.cn/GSC-A/#/) (22) and Genomics of Drug Sensitivity in Cancer (GDSC) databases.

### Immunohistochemical Staining and Evaluation

We performed IHC staining on paraffinized tumor tissue microarray sections according to a previously published protocol (23). Human KIRC tissue microarray (5-μm thick; cat. no. SP013-U1, Expectlab, Qingdao, Shandong, China) and human LIHC tissue microarray (5-μm thick; cat. no. SP014-T2, Expectlab) sections were deparaffinized, rehydrated, and subjected to antigen retrieval, blocking (3% H_2_O_2_, 5% goat serum), and incubation with primary rabbit anti-human TRPM8 antibody (1:200; cat. no. PB0882, Boster Biological Technology Co., Ltd., Wuhan, China) overnight at 4°C. Visualization of the antigen-antibody complexes was performed with an AEC kit (cat. no. ZLI-9036, Beijing Zhongshan Jinqiao Biotechnology Co., Ltd.), based on biotin-streptavidin-peroxidase complexation. Integrated IHC staining intensity was used to evaluate the TRPM8 level in each human tumor tissue point. The images were analyzed using Image Tool II software (University of Texas Health Science Center, San Antonio, TX, USA). The staining intensity of TRPM8 was evaluated using a grayscale ranging from 0 to 255.

### Ethics Statement

The tissue microarrays used in this study are commercially available, which waived the need for ethical approval from the Ethics Committee of Jiangnan University.

### Statistical Analysis

Bioinformatic data were analyzed using R v 4.0.3. Statistical analysis of IHC results was performed using SPSS (statistical package for the social sciences) software (SPSS Inc., Chicago, IL, Version 17.0). Data were expressed as means ± SEM and analyzed using Student’s *t*-test for independent samples. P < 0.05 was considered significant.

## Results

### TRPM8 Levels in Cancer Tissues

**Figure 1:**
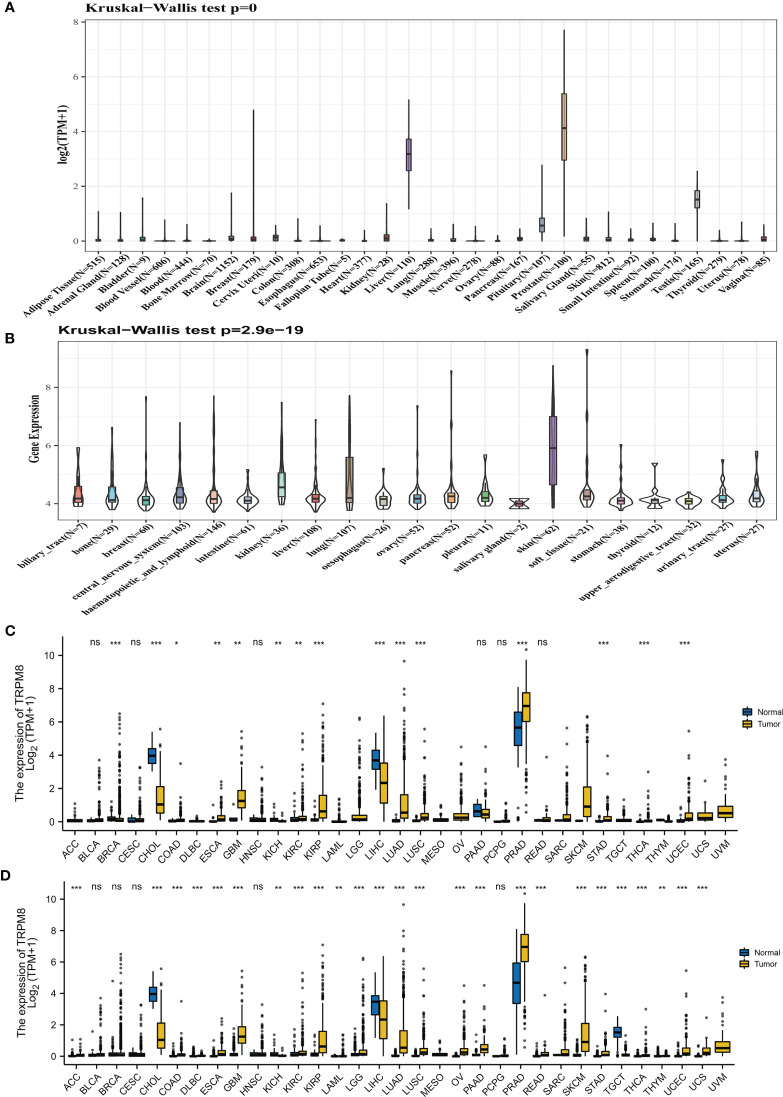
TRPM8 levels in tumor tissues. **(A)** TRPM8 levels in 31 cancers, from GTEx. **(B)** TRPM8 levels in 21 cell lines, from CCLE. **(C)** TRPM8 levels in 33 pairs of tumor and non-tumor tissues, from TCGA. *P < 0.05, **P < 0.01, and ***P < 0.001. **(D)** TRPM8 differential expression in 33 cancers, from integrated GTEx and TCGA data, ns, no significance, *P < 0.05, **P < 0.01, ***P < 0.001.

The TRPM8 mRNA levels derived from the GTEx datasets for 31 cancer types are shown in **Figure 1A**, while **Figure 1B** illustrates TRPM8 levels in CCLE cell lines. The differences between tumor and adjoining control tissues (from TCGA) are seen in **Figure 1C**. The integration of the GTEx and TCGA data in 33 cancers (**Figure 1D**) indicates an elevation of TRPM8 levels in most solid tumors.

### Correlation of Clinical Phase and Molecular Subtype With TRPM8 mRNA Levels

**Figure 2:**
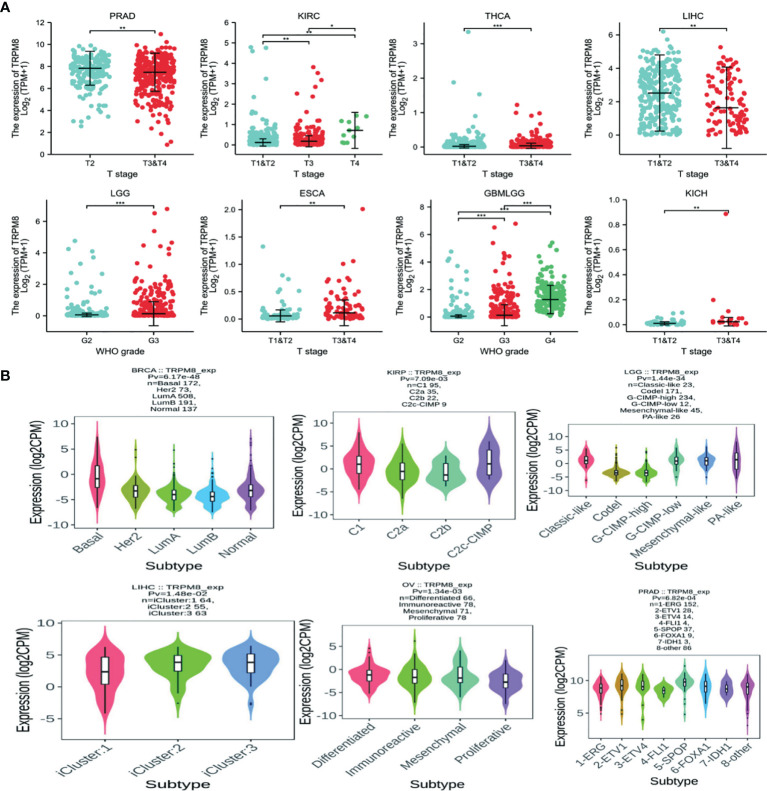
TRPM8 mRNA levels in different tumor types. **(A)** TRPM8 in relation to clinical stage, from TCGA,*P < 0.05, **P < 0.01, and ***P < 0.001. **(B)** TRPM8 in relation to molecular subtype, from TISIDB.

Significant differences in TRPM8 levels in relation to the clinical stage were seen in PRAD, KIRC, THCA, LIHC, LGG, ESCA, GBMLGG, and KICH (**Figure 2A**). TRPM8 levels also differed significantly according to molecular subtype in BRCA, KIRP, LGG, LIHC, OV, and PRAD (**Figure 2B**). (**Supplementary Figure S1A, S1B**).

### TRPM8 Gene Expression and Mutation

**Figure 3:**
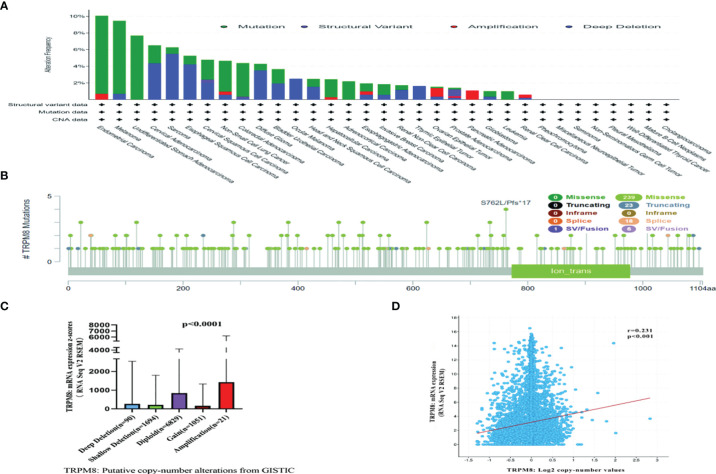
Mutation of TRPM8, assessed by cBioPortal. Mutation frequencies are shown with the type of mutation **(A)**, and site of mutation **(B)**. Association between TRPM8 copy number and mRNA level, shown by dot plot **(C)** and correlation plot **(D)**.

Both genetic and epigenetic factors influence tumor development (24). The frequency of mutation in TRPM8 was investigated using cBioPortal on 10967 samples from 32 studies. It was found that the highest mutation numbers were seen in UCEC, melanoma, and STAD, with TRPM8 representing approximately 7% of mutations (**Figure 3A**). Two hundred and eighty-seven mutation sites were observed, including 239 missense, 23 truncating, 18 splice, and 7 fusion variants (**Figure 3B**) over the 1100-residue length. Deletions were seen in over a third of tumors (**Figure 3C**), resulting in reduced mRNA expression. There was an association between TRPM8 copy number and mRNA level (**Figure 3D**). Using UALCAN, lower DNA methylation of TRPM8 was observed in BRCA, ESCA, COAD, HNSC, KIRC, LIHC, LUAD, LUSC, READ, SARC, TGCT, THCA, and UCEC tumors relative to controls. This indicates that elevated TRPM8 mRNA is likely to be caused by both genetic and epigenetic changes (**Supplementary Figure S2**).

### Association of TRPM8 With Prognosis

**Figure 4:**
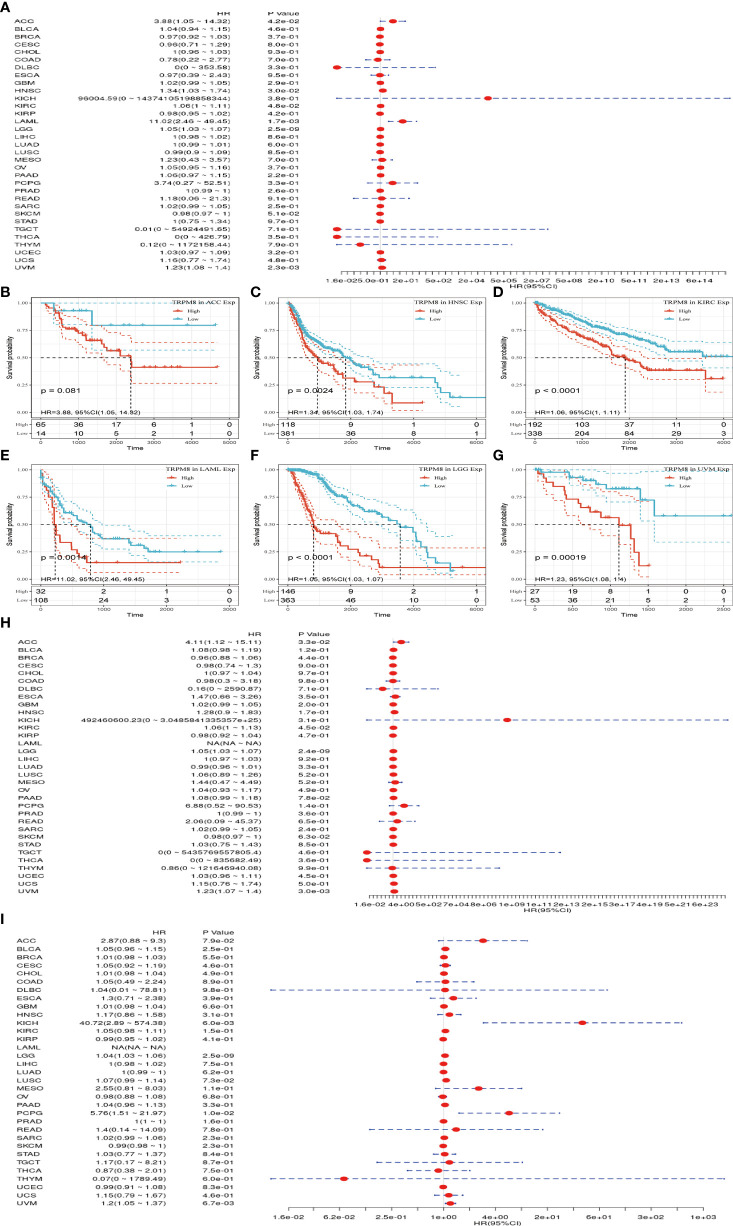
TRPM8 levels in relation to patient survival. **(A)** Association with OS in 33 cancer types. **(B-G)** Kaplan-Meier curves showing an association between TRPM8 and OS in the six most significantly associated tumors (ACC, HNSC, KIRC, LAML, LGG, and LIHC). **(H-I)** Association between TRPM8 and DSS and PFI.

Univariate analysis was performed to investigate the link between TRPM8 and OS in 33 tumors from TCGA. TRPM8 was significantly linked with OS in ACC (HR = 3.88, P = 0.042), HNSC (HR = 1.34, P = 0.002), KIRC (HR = 1.06, P < 0.001), LAML (HR = 11.02, P =0.001), LGG (HR = 1.05, P <0.001), and LIHC (HR = 1.23, P < 0.001) (**Figure 4A**). These findings indicate that TRPM8 is linked with poor outcomes, especially in LAML. The Kaplan-Meier curves for cancer types with significant TRPM8 association are shown in **Figures 4B–G**. **Figures 4H, I** shows the disease-free survival (DSS) and progression-free interval (PFI) which were determined to avoid bias from non-cancer mortality.

**Figure 5:**
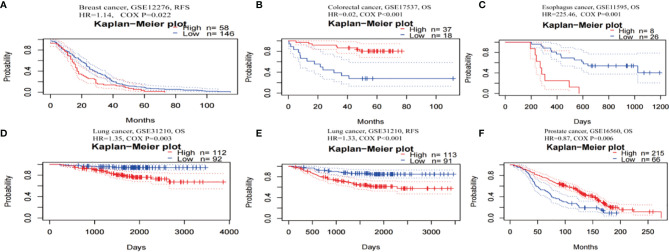
Kaplan-Meier survival curves for patients with high or low TRPM8 levels, from PrognoScan. **(A)** RFS (n = 204) in breast cancer cohort GSE12276. **(B)** OS (n = 55) in colorectal cancer cohort GSE17537. **(C)** OS (n = 34) in esophagus cancer cohort GSE11595. **(D, E)** OS (n = 204) and RFS (n = 204) in lung cancer cohort GSE31210. **(F)** OS (n = 281) in prostate cancer cohort GSE16560. DSS, disease-specific survival; OS, overall survival; RFS, relapse-free survival.

The prognostic worth of TRPM8 was then examined using PrognoScan. This showed significant value in five cancers, namely, colorectal, esophageal, breast, lung, and prostate cancers (**Figure 5**). TRPM8 adversely affected three tumor types, namely, breast (**Figure 5A**, RFS: Cox P = 0.022), esophageal (**Figure 5C**, OS: Cox P = 0.001), and lung (**Figures 5D, E**, OS: Cox P = 0.003; RFS: Cox P < 0.001) cancers but appeared to have a protective function in colorectal (**Figure 5B**, OS: Cox P <0.001) and prostate cancers (**Figure 5F**, OS: Cox P = 0.006). **Supplementary Table 1** contains detailed information on these cohorts.

Thus, TRPM8 overexpression was significantly linked with reduced survival in several tumors, suggesting its possible usefulness as a prognostic indicator.

### TRPM8 Association With Immune Infiltration

**Figure 6:**
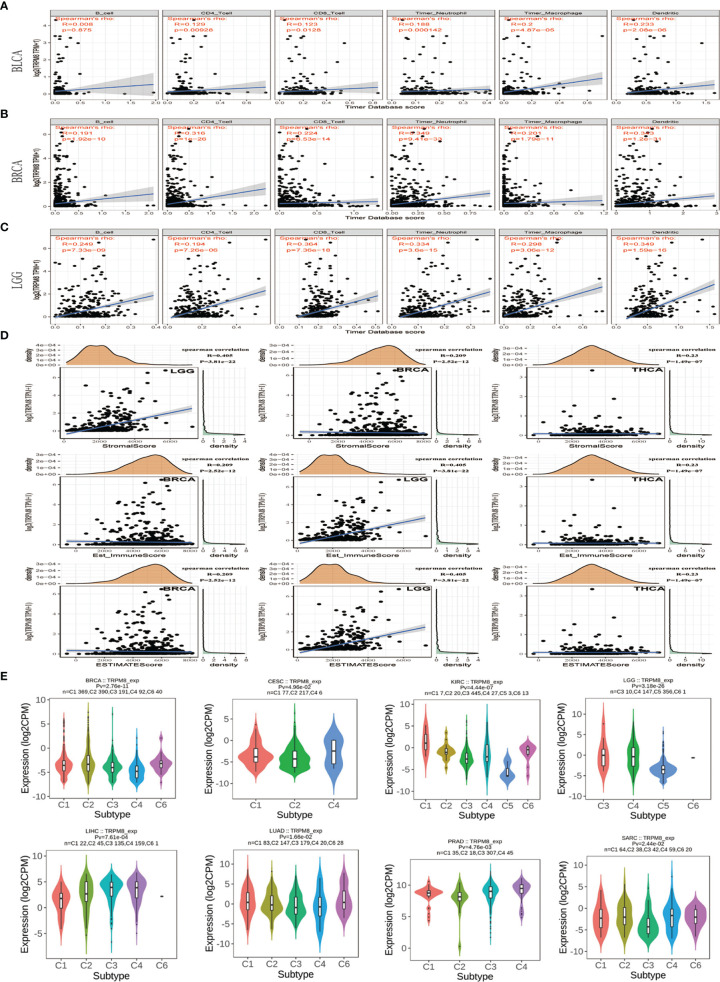
Associations between TRPM8 and immune infiltration in different tumors. **(A)** BLCA. **(B)** BRCA. **(C)** LGG. **(D)** Correlation between TRPM8 and immune score, stromal score, and estimate immune score. **(E)** TRPM8 mRNA levels in different immune subtypes in BRCA, CESC, KIRC, LGG, LIHC, LUAD, PRAD, and SARC *via* TISIDB. C1 (wound healing); C2 (IFN-gamma-dominant); C3 (inflammatory); C4 (lymphocyte-depleted); C5 (immunologically quiet); C6 (TGF-b-dominant).

TRPM8 levels were associated with the degree of immune infiltration in various tumors, including BLCA, BRCA, and LGG. As seen in **Figure 6A**, there were significant correlations with CD4+ T cells (R= 0.129, P < 0.01), CD8+ T cells (R= 0.123, P < 0.05), neutrophils (R = 0.188, P <0.001), macrophages (R = 0.2, P < 0.001), and dendritic cells (R = 0.233, P < 0.001) in BLCA. As seen in **Figures 6B, C**, TRPM8 was significantly associated with all six tumor-infiltrating cells, including B cells (R = 0.191, P < 0.001), CD4+ T cells (R = 0.316, P < 0.001), CD8+ T cells (R = 0.224, P < 0.001), neutrophils (R = 0.349, P < 0.001), macrophages (R = 0.201, P < 0.001), and dendritic cells (R = 0.343, P < 0.001) in BRCA (B), and with B cells (R = 0.249, P < 0.001), CD4+ T cells (R = 0.194, P < 0.001), CD8+ T cells (R = 0.364, P < 0.001), neutrophils (R = 0.334, P < 0.001), macrophages (R = 0.298, P < 0.001), and dendritic cells (R = 0.349, P < 0.001) in LGG (C). Furthermore, analysis of the immune and stromal scores using the R package “estimate” showed that the highest correlations between TRPM8 and immune score were LGG (R = 0.405, P < 0.001), BRCA (R = 0.209, P < 0.001), and THCA (R = 0.230, P < 0.001) (**Figure 6D**). The highest correlations with stromal score were seen in BRCA (R = 0.209, P < 0.001), LGG (R = 0.405, P < 0.001), and THCA (R =0.230, P < 0.001), and estimated immune scores were BRCA (R = 0.209, P < 0.001), LGG (R = 0.405, P <0.001) and THCA (R = 0.23, P < 0.001). Evaluation of molecular subtypes showed significance in the C1 (wound healing), C2 (IFN-γ-dominant), C3 (inflammatory), C4 (lymphocyte depleted), C5 (immunologically quiet), and C6 (TGF-β dominant) subtypes in BRCA, CESC, KIRC, LGG, LIHC, LUAD, PRAD, and SARC (**Figure 6E**). Other tumor subtypes did not differ significantly (**Supplementary Figure S3**). TRPM8 levels were highest in subtypes C6 in LUAD and BRCA, C4 in CESC and PRAD, and C3 in LGG. Different levels of expression may partly explain why TRPM8 appears to have different effects on prognosis in different cancers.

### Association Between TRPM8 Copy Number Variation and Immune Infiltrates

**Figure 7:**
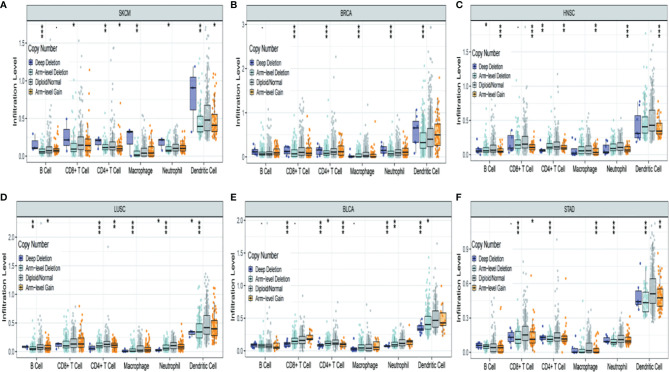
Association between TRPM8 copy number variation and immune infiltrates in **(A)** SKCM (skin cutaneous melanoma), **(B)** BRCA (breast invasive carcinoma), **(C)** HNSC (head and neck cancer), **(D)** LUSC (lung squamous cell carcinoma), **(E)** BLCA (bladder urothelial carcinoma), and **(F)** STAD (stomach adenocarcinoma). *P < 0.05, **P < 0.01, ***P < 0.001.

**Figure 7** shows the six most significant associations between TRPM8 copy number variation and immune infiltration in tumors. TRPM8 deletion was related to higher infiltration and was linked to markedly raised numbers of infiltrates in SKCM (**Figure 7A**). In contrast, TRPM8 deletion was linked to lower infiltration in BRCA (**Figure 7B**), HNSC (**Figure 7C**), LUSC (**Figure 7D**), BLCA (**Figure 7E**), and STAD (**Figure 7F**). These results may suggest reasons for TRPM8 changes in prognostic prediction.

### Association of TRPM8 With Immune Neoantigens and Checkpoint Genes

**Figure 8:**
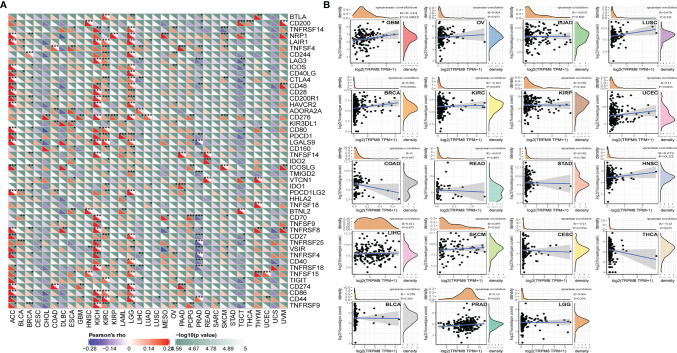
Relationships between TRMP8 and immune neoantigens and checkpoint genes. **(A)** Relationship between TRPM8 and checkpoint gene levels. *P < 0.05, **P < 0.01, and ***P < 0.001. **(B)** Relationship between TRPM8 and neoantigen numbers in 19 tumor types.

The levels of over 40 checkpoint genes were examined in different tumor types (**Figure 8A**). TRPM8 was found to be positively related to checkpoint gene levels in a number of tumors, including ACC, KIRC, KICH, and LGG. TRPM8 levels were also positively associated with neoantigen number in BRCA and PRAD (R = 0.10, P =0.0093; R = 0.14, P = 0.024, respectively) (**Figure 8B**).

### Association of TRPM8 Expression With TMB and MSI

**Figure 9:**
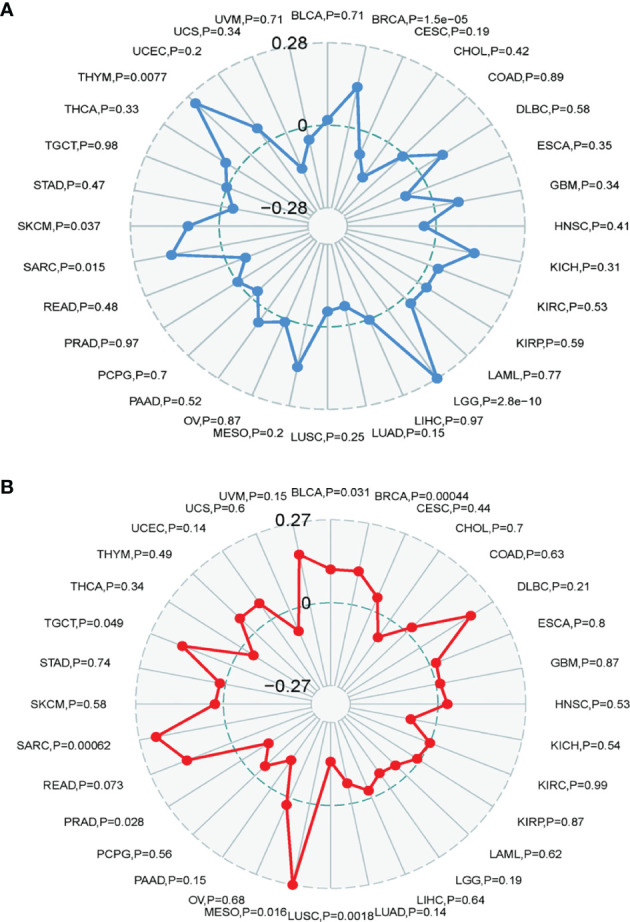
Correlation between TRMP8 and TMB and MSI. **(A)** TRPM8 correlation with TMB. **(B)** TRPM8 correlation with MSI.

The TMB represents the number of mutations in a cancer cell. TRPM8 levels were associated with TMB in THYM, BRCA, LGG, SARC, and SKCM (**Figure 9A**). TRMP8 was also associated with MSI in SARC, TGCT, BLCA, BRCA, and MESO, and negatively correlated with LUSC and PRAD (**Figure 9B**).

### Influence of TRPM8 on DNA MMR Genes and Methyltransferases

**Figure 10:**
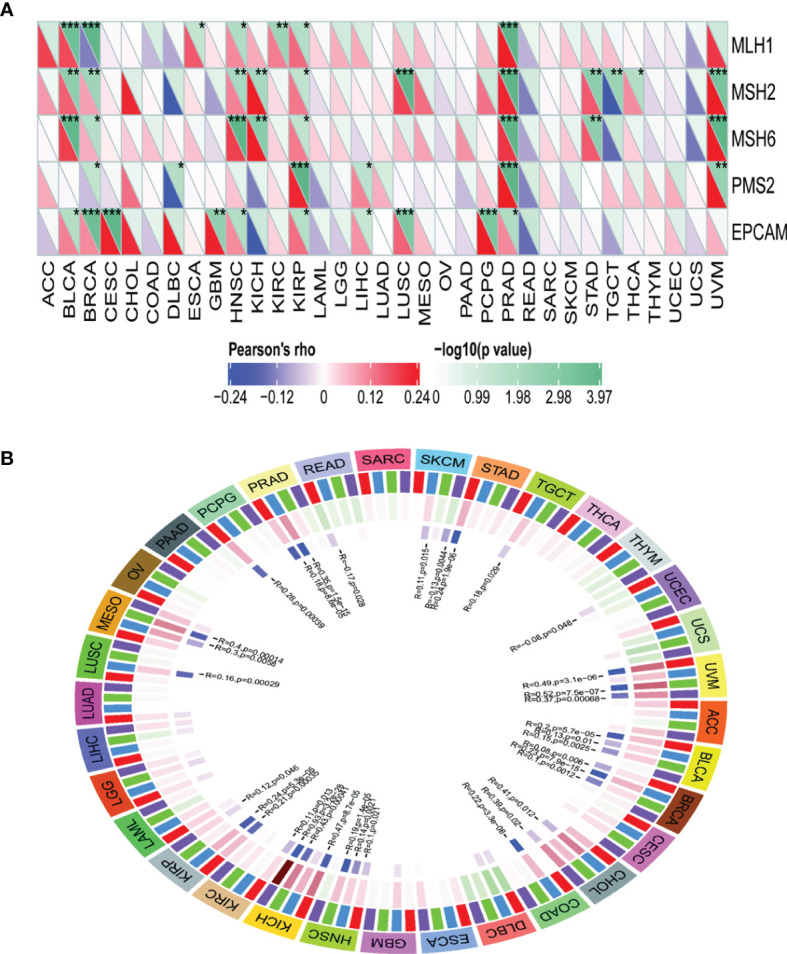
Correlations between TRPM8 and DNA MMR genes and methyltransferases. **(A)** Correlation between TRPM8 and five MMRs. **(B)** Correlation between TRPM8 and four methyltransferases. DNMT1 is colored red, DNMT2 blue, DNMT3a green, and DNMT3b purple. *P < 0.05, **P < 0.01, ***P < 0.001.

TRPM8 levels were positively correlated with most MMR genes in BLCA, BRCA, HNSC, KIRP, PRAD, and UVM (**Figure 10A**), as well as with the levels of methyltransferases in UVM, BLCA, BRCA, HNSC, KICH, and KIRP (**Figure 10B**), indicating that TRPM8 can modulate tumorigenesis through epigenetic means. 

### Enrichment Analysis of TRPM8-Associated Partners

**Figure 11:**
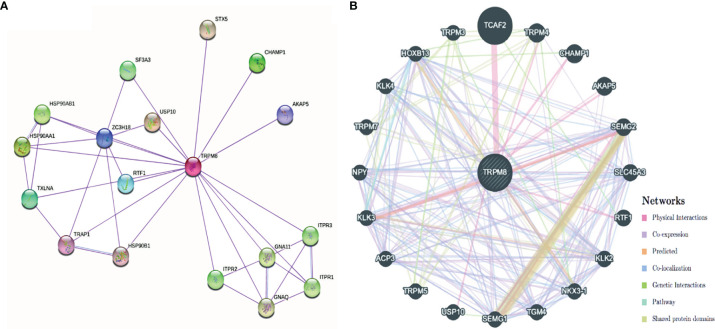
PPI networks of TRPM8 and its interacting protein partners. **(A)** Network constructed by STRING. **(B)** Network constructed by GeneMANIA.

The PPI network created by STRING showed associations between TRPM8 and HSP90, Ubiquitinase USP10, and inositol triphosphate receptors (ITPR1, ITPR2, ITPR3), amongst others (**Figure 11A**). The network constructed by GeneMANIA showed interaction with TCAF2 (TRPM8 channel associated factor 2) (**Figure 11B**), which is involved in the regulation of TRPM8 activity in the membrane. Interactions with TRPM4 and CHAMP1 were also predicted.

**Figure 12:**
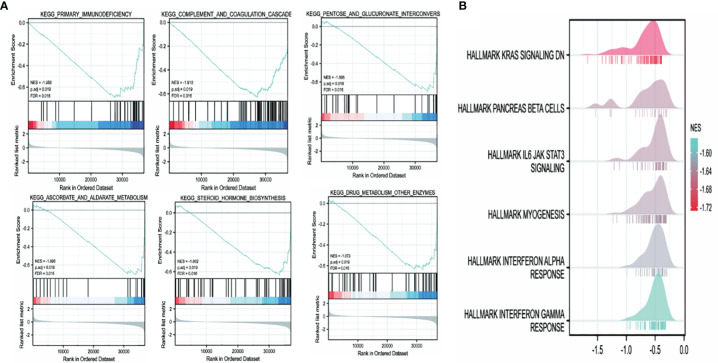
TRPM8 enrichment pathways predicted by KEGG and Hallmark. **(A)** Top six pathways identified by KEGG. **(B)** Top six pathways identified by Hallmark.

The high and low TRPM8 expression groups were investigated for KEGG and Hallmark pathway enrichment (listed in **Supplementary Tables 2** and **3**). Enrichments were seen (**Figure 12**) in the “primary immunodeficiency pathway” and “KRAS signaling pathway”. KEGG pathways included “complement and coagulation cascades”, “pentose and glucuronate interconversions”, “steroid hormone biosynthesis”, and “cytokine-cytokine receptor interaction”. “IL6/JAK/STAT3 signaling”, “Interferon (IFN) cell signaling pathway”, “inflammatory response”, “TNFalpha/NF-κB pathway”, “angiogenesis”, “epithelial-mesenchymal transition”, “hypoxia” and others were identified by Hallmark. Thus, TRPM8 appears to be involved in pathways associated with tumor physiology and immunity.

### Association Between TRPM8 and Drug Sensitivity

**Figure 13:**
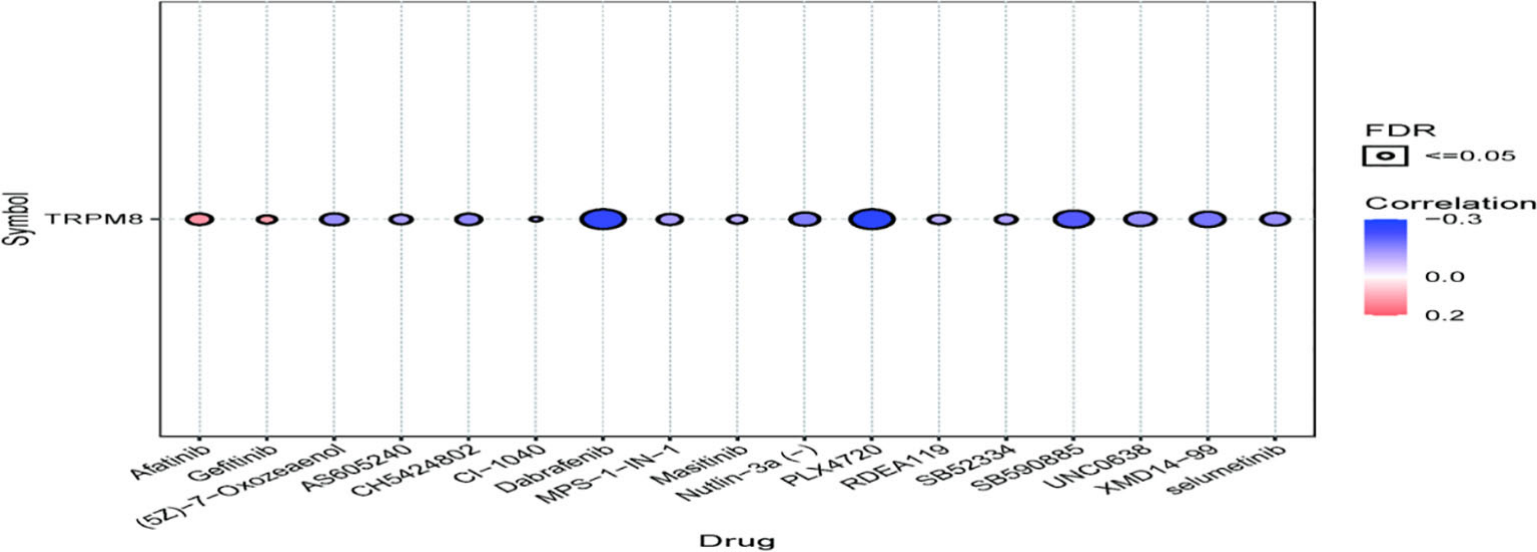
Association between TRPM8 and drug (top 30) sensitivity, shown by GDSC analysis.

TRPM8 was observed to be linked to the response to Afatinib, Dabrafenib, and PLX4720, shown by GDSC (**Figure 13**), suggesting the application of these findings in treatment.

### Preliminary Experimental Verification of TRPM8 in KIRC and LIHC

**Figure 14:**
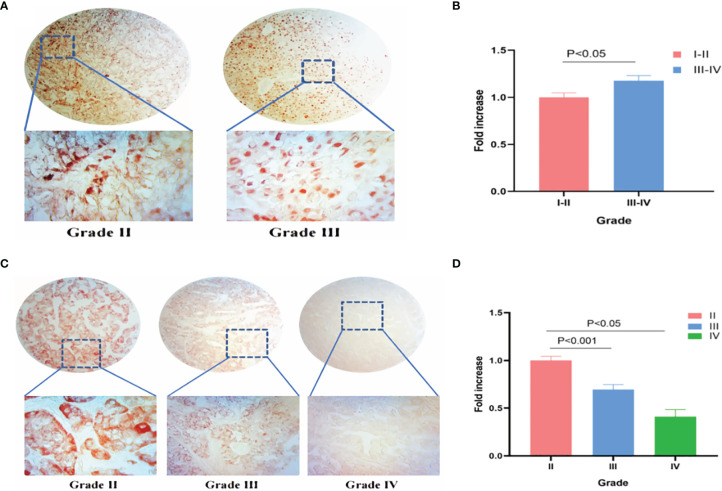
TRPM8 expression in human KIRC and LIHC tissues. **(A)** IHC staining for TRPM8 in KIRC tissues. **(B)** Comparative statistical analysis of TRPM8 levels measured as staining intensity in different grades of KIRC tissues. **(C)** IHC staining for TRPM8 in LIHC tissues. **(D)** Comparative statistical analysis of TRPM8 levels measured as staining intensity in different grades of LIHC tissues.

IHC was used to verify TRPM8 levels in KIRC and LIHC tissues. 90 grade I−II and 42 grade III−IV KIRC tissues were examined, finding greater TRPM8 expression in grade III−IV material (p < 0.05) (**Figures 14A, B**). In LIHC samples (88 grade II, 53 grade III, and 3 grade IV), however, it was observed that TRPM8 levels were reduced in the higher grades, with lower TRPM8 expression seen in both grades III and IV compared with grade II (p < 0.001 vs. grade II for grade III, and p < 0.05 vs. grade II for grade IV) (**Figures 14C, D**).

## Discussion

The TRP family of ion channels was first identified in Drosophila (25, 26) and consists of six subfamilies of which the melastatin subfamily (TRPM) is linked to tumor progression (27–29). TRPM8 is a six-pass transmembrane protein containing 1104 residues with a molecular weight of 128 kDa (30). The channel is formed between the S5 and S6 domains (31).

In mammals, TRPM8 was first described in sensory neurons connected with cold perception (32) and was subsequently found in many other cell types, including tumor cells (33). Intriguingly, TRPM8 appears to have opposite functions according to the tumor type; it is overexpressed in breast cancers and its silencing leads to reduced tumor growth (34). A similar situation was reported in oral squamous carcinoma (35). We observed pro-cancerous functions of TRPM8 in ACC, COAD, DLBC, ESCA, GBM, KIRC, KIRP, LGG, LUAD, LUSC, OV, PAAD, PRAD, READ, SKCM, STAD, THCA, THYM, UCEC, and UCS, but anti-tumor actions in CHOL, HNSC, KICH, LAML, LIHC, and testicular germ cell tumors. Cisplatin induction of TRPM8 is reported to lead to calcium influx in testicular cancer, reducing tumor growth (36). These findings indicate that TRPM8 function varies with the cancer type.

Both genetics and epigenetics play crucial roles in cancer. For example, mutations in PD-L1 affect the protein’s structure, expression, and function (37, 38). Amplification of JAK2/PD-L1/PD-L2 on chromosome 9 results in overexpression and alterations in immune checkpoints (39, 40). The cellular localization of PD-L1 may be dependent on transcriptional regulation. In TRPM8, both genetic polymorphism and differential methylation of the promoter regions may alter the protein expression. The abnormal increase of TRPM8 mRNA expression in some cancers is likely a result of lower DNA methylation levels. In addition to its function in immune modulation, our results showed that TRPM8 is involved in a variety of pathways, both signaling and biosynthetic, shown by KEGG enrichment in “IL6/JAK/STAT3 signaling”, “interferon (IFN) cell signaling pathway”, “inflammatory response”, “TNFalpha/NF-κB pathway”, “angiogenesis”, “epithelial-mesenchymal transition”, and “hypoxia”. All these pathways were associated with the “high expression” TRPM8 group. It thus appears that TRPM8 overexpression activates these pathways, with TRPM8 stimulating angiogenesis, tumor viability, and growth.

Other genes have also been shown to function differently according to cell and cancer type (41). TRPM8 may thus have different prognostic values in different tumors, and perhaps even between different subtypes of the same tumor. Tumor heterogeneity is recognized as a serious challenge to effective cancer therapy (42, 43). It is possible that TRPM8 may promote tumorigenesis in “cold tumors” such as BRCA-luminal A/B while in “hot tumors” such as BRCA-basal, TRPM8 may modulate immunosuppression through pathway regulation. It is thus possible that TRPM8 may promote tumor heterogeneity. TRPM8 supports immune escape by esophageal cancer cells through induction of PD-L1 (44). Apart from CD8+ T cells, we found significant infiltration by other immune cells especially in BLCA, BRCA, and LGG, indicating the strong connection between TRPM8 and immunity.

Despite its integration of multiple datasets, there are limitations to this study. First, much of the sequencing and microarray data were obtained from gene expression data and systematic bias could have arisen from the cellular expression of immune cell biomarkers. To avoid this, large sample numbers and analyses are required for individual subgroups (45, 46). Second, the databases lack data on posttranslational modifications which may conceivably affect the functioning of TRPM8. Third, this study was a bioinformatic analysis with limited empirical verification; further studies are required. Fourth, despite the correlations obtained between TRPM8 and immune infiltration and patient outcomes, we have no direct proof that TRPM8 influences outcomes by immune modulation. This requires further prospective evaluation in cancer patients.

To summarize, we used an integrated bioinformatic approach which found that high levels of TRPM8 may modulate immune infiltration and influence patient outcomes in many cancers. This suggests that TRPM8 may be useful as a biomarker for prognostic prediction and suggests directions for therapeutic development.

## Data Availability Statement

The original contributions presented in the study are included in the article/**Supplementary Material**. Further inquiries can be directed to the corresponding author.

## Ethics Statement

The data in this study are from public bioinformatics databases, which waived the need for ethical approval from the Ethics Committee of Jiangnan University. The tissue microarrays used in this study are commercially available, which waived the need for ethical approval from the Ethics Committee of Jiangnan University.

## Author Contributions

WZ drafted the manuscript and was responsible for bioinformatical data acquisition; X-yQ and QL participated in immunohistochemical data analysis; C-mQ assisted in literature search and data processing using software; CC and Y-qS revised the manuscript; W-jZ organized writing and data visualization of the article, undertook immunohistochemical staining of the tissue microarray, and corrected and finalized the manuscript. All authors read and approved the manuscript and agreed to take responsibility for all aspects of the study. All authors contributed to the article and approved the submitted version.

## Funding

This work was supported by grants from Guizhou Nursing Vocational College Foundation (gzhlyj2021-02) and Science and Technology Foundation of Guizhou Provincial Health Committee (gzwkj2022-518), Jiangsu Province Shuangchuang Talent Plan (Grant No. JSSCRC 2021533).

## Conflict of Interest

The authors declare that the research was conducted in the absence of any commercial or financial relationships that could be construed as a potential conflict of interest.

## Publisher’s Note

All claims expressed in this article are solely those of the authors and do not necessarily represent those of their affiliated organizations, or those of the publisher, the editors and the reviewers. Any product that may be evaluated in this article, or claim that may be made by its manufacturer, is not guaranteed or endorsed by the publisher.
