# Supplementary material for: Comprehensive Pan-Cancer Analysis of TRPM8 in Tumor Metabolism and Immune Escape
Source: Front Oncol. 2022 Jun 30;12:914060. doi: 10.3389/fonc.2022.914060 (PMC9281503; doi:10.3389/fonc.2022.914060)
Supplement: Supplementary file 1 [file DataSheet_1.docx]

**Comprehensive Pan-Cancer analysis of TRPM8 in tumor metabolism and immune escape**

Wei Zhang^1, 2^, Xin-yu Qiao^1^, Qian Li^1^, Chun Cui^3^, Chen-meng Qiao^3^, Yan-qin Shen^3^, Wei-jiang Zhao^1*^

1. Cell Biology Department, Wuxi School of Medicine, Jiangnan University, Wuxi, 214122, Jiangsu, China

2. Department of Pathogen Biology, Guizhou Nursing Vocational College, Guiyang, China

3. Department of Neurodegeneration and Neuroinjury, Wuxi School of Medicine, Jiangnan University, Wuxi, 214122, Jiangsu, China.

Correspondence to: Weijiang Zhao. Cell Biology Department, Wuxi School of Medicine, Jiangnan University, Wuxi, Jiangsu, 214000, China

E-mail: weijiangzhao@jiangnan.edu.cn

**This file includes:** Supplementary Figures S1 to S3

Supplementary Table 1 to Table 3

**Figure S1** **The transcription levels of TRPM8 in human cancers.**

**(A)** No difference was found between TRPM8 expression and stages in these cancers. **(B)** No difference was found between TRPM8 expression and molecular subtypes via TISIDB.

**Figure S2 The differential promoter DNA methylation status of TRPM8 between cancer and adjacent normal tissues by using UALCAN.** The abnormal increase of TRPM8 mRNA expression in some cancers is likely a result of lower DNA methylations levels.

**Figure S3 Correlation analysis between TRPM8 expression and molecular typing of immune subtypes by using TISIDB .** No difference was found between TRPM8 expression and molecular typing of immune subtypes via TISIDB.

**Figure S1**


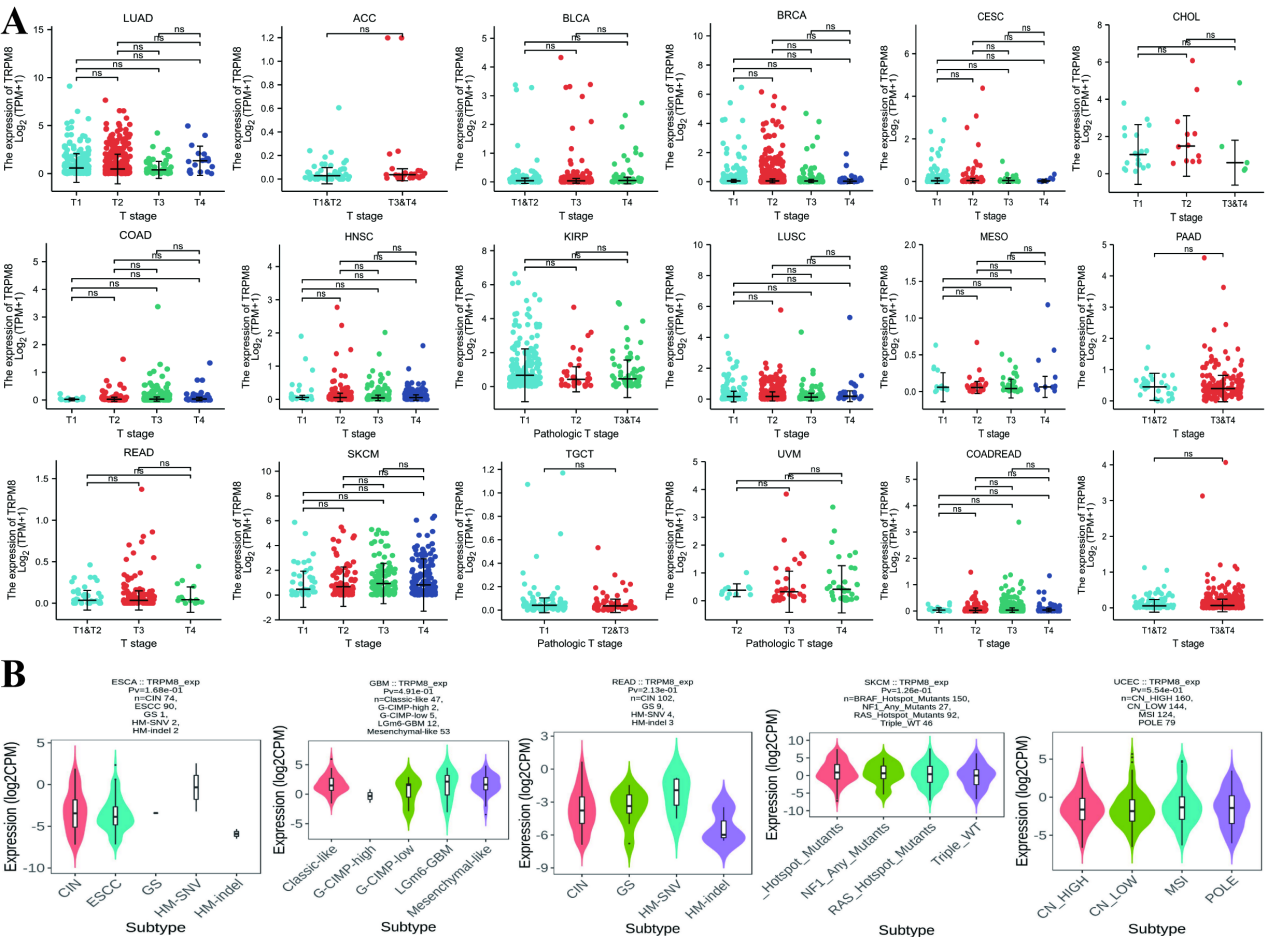


**Figure S2**

**
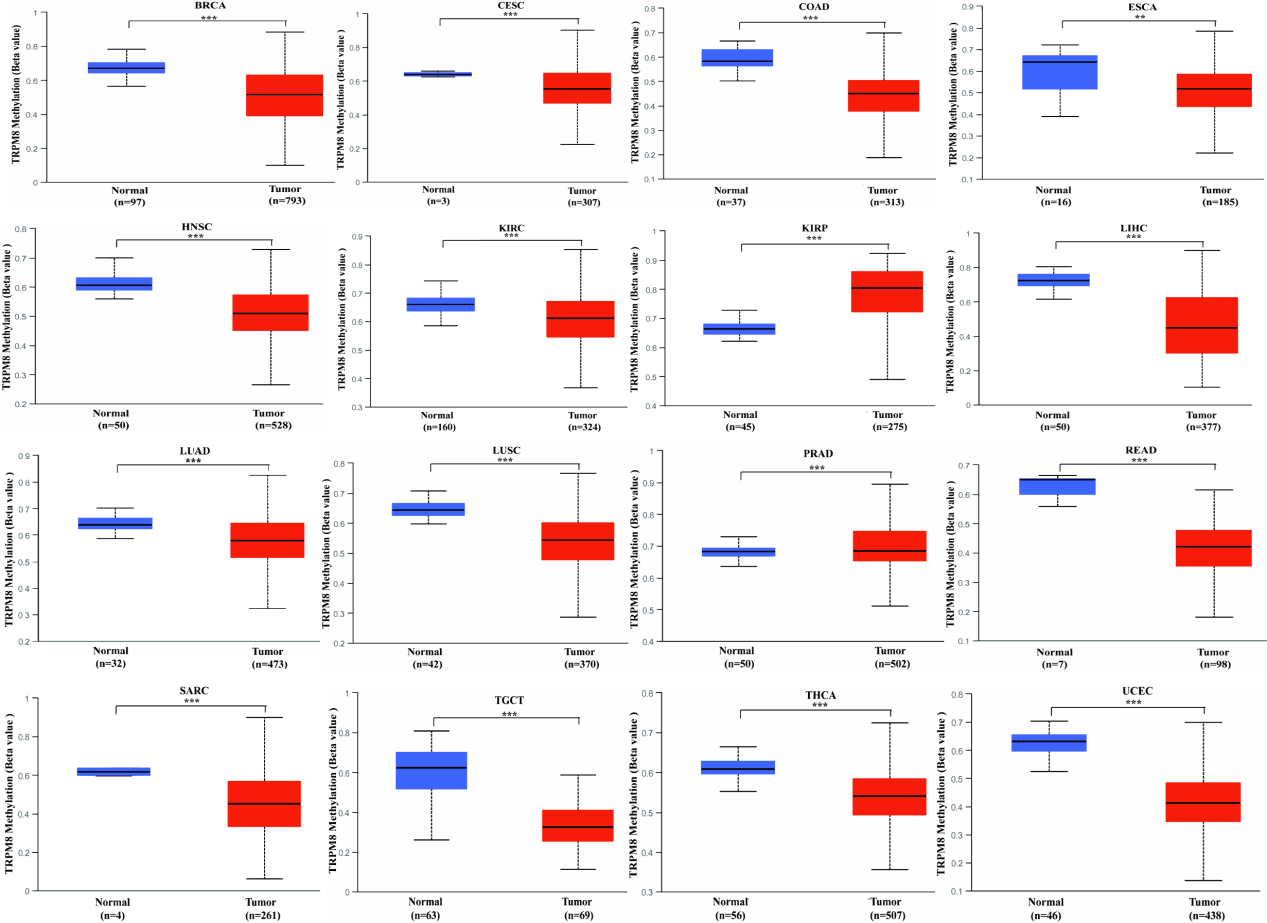
**

**Figure S3**

**
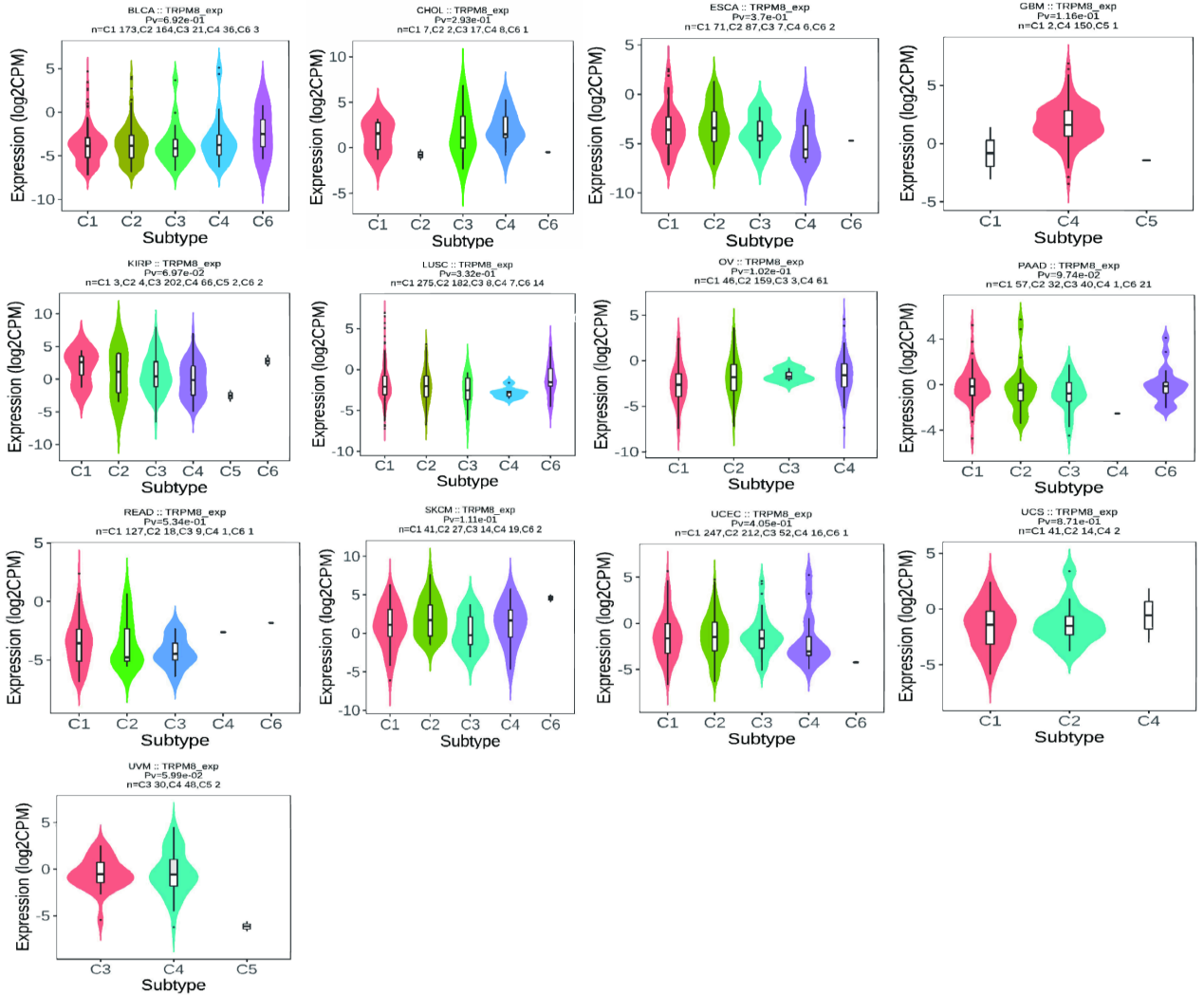
**

**Supplementary Table 1**

**TRPM8 Correlates With Immune Infiltration and Impacts Patient Prognosis_ A Pan-Cancer Analysis**

| ID_NAME | DATASET | CANCER TYPE | SUBTYPE | ENDPOINT | N | CUTPOINT | HR [95% CI-low CI-upp] | COX P-VALUE | Sig |
| --- | --- | --- | --- | --- | --- | --- | --- | --- | --- |
| TRPM8 | GSE12276 | Breast cancer |  | Relapse Free Survival | 204 | 0.715686 | 1.14 [1.02 - 1.27] | 0.0229909 | * |
| TRPM8 | GSE17537 | Colorectal cancer |  | Overall Survival | 55 | 0.327273 | 0.02 [0.00 - 0.18] | 0.000492732 | *** |
| TRPM8 | GSE11595 | Esophagus cancer | Adenocarcinoma | Overall Survival | 34 | 0.764706 | 225.46 [8.47 - 6004.05] | 0.00121377 | ** |
| TRPM8 | GSE31210 | Lung cancer | Adenocarcinoma | Overall Survival | 204 | 0.45098 | 1.35 [1.11 - 1.64] | 0.0030877 | ** |
| TRPM8 | GSE31210 | Lung cancer | Adenocarcinoma | Relapse Free Survival | 204 | 0.446078 | 1.33 [1.14 - 1.55] | 0.000357953 | *** |
| TRPM8 | GSE16560 | Prostate cancer |  | Overall Survival | 281 | 0.234875 | 0.87 [0.78 - 0.96] | 0.00696788 | ** |

**Supplementary Table 2 Top 15 KEGG pathways.**

| **Term** | **ES** | **NES** | **pvalue** | **qvalues** |
| --- | --- | --- | --- | --- |
| KEGG_PRIMARY_IMMUNODEFICIENCY | -0.6957 | -1.9577 | 0.0011 | 0.0160 |
| KEGG_COMPLEMENT_AND_COAGULATION_CASCADES | -0.6307 | -1.9177 | 0.0010 | 0.0160 |
| KEGG_PENTOSE_AND_GLUCURONATE_INTERCONVERSIONS | -0.6987 | -1.8979 | 0.0011 | 0.0160 |
| KEGG_ASCORBATE_AND_ALDARATE_METABOLISM | -0.7097 | -1.8962 | 0.0011 | 0.0160 |
| KEGG_STEROID_HORMONE_BIOSYNTHESIS | -0.6347 | -1.8816 | 0.0010 | 0.0160 |
| KEGG_DRUG_METABOLISM_OTHER_ENZYMES | -0.6379 | -1.8729 | 0.0010 | 0.0160 |
| KEGG_METABOLISM_OF_XENOBIOTICS_BY_CYTOCHROME_P450 | -0.6020 | -1.8304 | 0.0010 | 0.0160 |
| KEGG_CYTOKINE_CYTOKINE_RECEPTOR_INTERACTION | -0.5616 | -1.8304 | 0.0010 | 0.0160 |
| KEGG_INTESTINAL_IMMUNE_NETWORK_FOR_IGA_PRODUCTION | -0.6314 | -1.8263 | 0.0011 | 0.0160 |
| KEGG_ALLOGRAFT_REJECTION | -0.6465 | -1.8192 | 0.0011 | 0.0160 |
| KEGG_RETINOL_METABOLISM | -0.5840 | -1.7655 | 0.0010 | 0.0160 |
| KEGG_STARCH_AND_SUCROSE_METABOLISM | -0.6002 | -1.7622 | 0.0010 | 0.0160 |
| KEGG_FOLATE_BIOSYNTHESIS | -0.7684 | -1.7502 | 0.0012 | 0.0160 |
| KEGG_GRAFT_VERSUS_HOST_DISEASE | -0.6157 | -1.7384 | 0.0011 | 0.0160 |
| KEGG_PORPHYRIN_AND_CHLOROPHYLL_METABOLISM | -0.6065 | -1.7304 | 0.0011 | 0.0160 |

**Supplementary Table 3 Top 15 Hallmark pathways.**

| **Term** | **ES** | **NES** | **pvalue** | **FDR** |
| --- | --- | --- | --- | --- |
| HALLMARK_KRAS_SIGNALING_DN | -0.5375 | -1.7270 | 0.0010 | 0.0023 |
| HALLMARK_PANCREAS_BETA_CELLS | -0.5766 | -1.6449 | 0.0021 | 0.0044 |
| HALLMARK_IL6_JAK_STAT3_SIGNALING | -0.5312 | -1.6425 | 0.0010 | 0.0023 |
| HALLMARK_MYOGENESIS | -0.5088 | -1.6347 | 0.0010 | 0.0023 |
| HALLMARK_INTERFERON_ALPHA_RESPONSE | -0.5212 | -1.6225 | 0.0010 | 0.0023 |
| HALLMARK_INTERFERON_GAMMA_RESPONSE | -0.4879 | -1.5684 | 0.0010 | 0.0023 |
| HALLMARK_INFLAMMATORY_RESPONSE | -0.4801 | -1.5425 | 0.0010 | 0.0023 |
| HALLMARK_TNFA_SIGNALING_VIA_NFKB | -0.4772 | -1.5339 | 0.0010 | 0.0023 |
| HALLMARK_EPITHELIAL_MESENCHYMAL_TRANSITION | -0.4731 | -1.5207 | 0.0010 | 0.0023 |
| HALLMARK_COMPLEMENT | -0.4669 | -1.5008 | 0.0010 | 0.0023 |
| HALLMARK_ANGIOGENESIS | -0.5129 | -1.4335 | 0.0372 | 0.0521 |
| HALLMARK_KRAS_SIGNALING_UP | -0.4262 | -1.3698 | 0.0060 | 0.0116 |
| HALLMARK_IL2_STAT5_SIGNALING | -0.4140 | -1.3300 | 0.0080 | 0.0144 |
| HALLMARK_APOPTOSIS | -0.4121 | -1.3158 | 0.0160 | 0.0252 |
| HALLMARK_HYPOXIA | -0.4042 | -1.2985 | 0.0130 | 0.0219 |
